# A systematic review: impact of dry needling, isometric, and eccentric exercises on pain and function in individuals with patellar tendinopathy

**DOI:** 10.3389/fresc.2024.1263295

**Published:** 2025-07-01

**Authors:** Faiza Sharif, Ashfaq Ahmad, Syed Amir Gilani, Dania Mahmood

**Affiliations:** ^1^ University Institute of Physical Therapy, University of Lahore, Lahore, Pakistan; ^2^ University of Lahore, Lahore, Pakistan

**Keywords:** patellar tendinopathy, exercise, pain, dry needling (DN), function

## Abstract

This systematic review aims to evaluate the existing body of evidence and provide a comprehensive analysis of the effects of dry needling, isometric, and eccentric exercises on pain severity and functional impairment in individuals diagnosed with patellar tendinopathy. The review was conducted in strict accordance with the Preferred Reporting Items for Systematic Reviews and Meta-Analyses (PRISMA) guidelines. Databases including the Cochrane Library, PubMed, PEDro, and Google Scholar were systematically searched for relevant literature from 2010 to August 2022. The therapeutic effects of dry needling, isometric exercises, and eccentric exercises on patellar tendinopathy were critically assessed. The risk of bias was determined using the McMaster Critical Review Form for Quantitative Studies, while the level of evidence and recommendation grades were assigned based on the Oxford Centre for Evidence-Based Medicine guidelines.

The primary outcomes of interest were pain severity and functional impairment, particularly among elite volleyball and basketball athletes suffering from patellar tendinopathy. The systematic review incorporated nine clinical trials, including two on dry needling, four on isometric exercises, and three on eccentric exercises. The quality of the included studies ranged from 70% to 93%, as evaluated through the McMaster Critical Review Form. The two dry needling trials were deemed to be of high quality (93%), while the four isometric trials were classified as moderate quality (75%). Similarly, the three trials on eccentric exercises were rated at an average of 78%, indicating moderate quality.

Of the nine trials, six were randomized controlled trials, with a 1B level of evidence (Grade A recommendation), while three trials, comprising two cross-over trials and one controlled clinical trial, were categorized as 2B evidence (Grade B recommendation). The findings from this review suggest that dry needling and eccentric exercises are more effective for long-term pain reduction and improvement in knee function, whereas isometric exercises appear to provide greater benefit for short-term pain relief, particularly during athletic activity. Although physical therapy-based interventions show promise for sustained management, the limited data underscores the need for further high-quality research in this domain.

**Systematic Review Registration:**

www.crd.york.ac.uk/PROSPERO/display_record.asp?ID=CRD42022360057, identifier (CRD42022360057).

## Introduction/background

Patellar tendinopathy, commonly known as “jumper's knee,” is a chronic overuse injury affecting the patellar tendon. This condition is particularly prevalent among athletes involved in sports requiring repetitive jumping and landing, such as volleyball and basketball. Understanding the etiology, pathophysiology, and clinical presentation of patellar tendinopathy is crucial for developing effective treatment strategies (1, 2).

Patellar tendinopathy typically results from repetitive mechanical overload on the patellar tendon. This overload leads to microtrauma, which, when coupled with insufficient recovery time, initiates a degenerative process within the tendon. The pathophysiology involves several stages: initial inflammatory response, failed healing response with increased production of type III collagen, neovascularization and nerve ingrowth, disorganization of collagen fibres, and eventual tendon degeneration and weakening. These changes result in pain, reduced function, and decreased athletic performance (3).

Patients with patellar tendinopathy typically present with localized pain at the inferior pole of the patella, pain exacerbated by activities involving knee extension, particularly jumping and landing, a gradual onset of symptoms that often worsen over time, stiffness after periods of inactivity, and decreased strength and performance in sport-specific activities. Diagnosis is primarily clinical, often supported by imaging techniques such as ultrasound or MRI to assess the extent of tendon degeneration (4).

Treatment for patellar tendinopathy can be broadly categorized into conservative and surgical approaches. Conservative treatments include rest and activity modification, physical therapy (which includes eccentric exercises, isometric exercises, and dry needling), non-steroidal anti-inflammatory drugs (NSAIDs), extracorporeal shock wave therapy (ESWT), platelet-rich plasma (PRP) injections, and corticosteroid injections (though these are controversial due to potential long-term negative effects) (5, 6). Surgical options include arthroscopic debridement, open tenotomy and repair, and ultrasound-guided percutaneous tenotomy. While surgical interventions can be effective in recalcitrant cases, they are typically considered only after conservative measures have failed (7).

Among the available treatment options, physical therapy-based interventions offer several distinct advantages for athletes with patellar tendinopathy. These advantages include their non-invasive nature, as physical therapy treatments do not require incisions or lengthy recovery periods, minimizing the risk of complications and allowing for a quicker return to sport. Many physical therapy protocols also allow athletes to continue modified training regimens, maintaining overall fitness and reducing deconditioning during the rehabilitation process (8). Physical therapy carries a significantly lower risk of complications compared to surgical interventions or injections, which can lead to adverse effects such as infection, scarring, or tendon weakening. Additionally, physical therapy is typically more cost-effective compared to surgical procedures or repeated injection therapies, making it a more accessible option for many athletes (9).

Physical therapy interventions can also identify and correct biomechanical factors contributing to tendon overload, potentially reducing the risk of recurrence. Furthermore, targeted exercises and manual therapies can stimulate tendon remodelling and improve load tolerance, addressing the underlying pathophysiology of the condition (10). Physical therapy protocols can be easily modified to suit an athlete's specific needs, sport demands, and stage of rehabilitation, offering versatility and individualization. Given these advantages, there is growing interest in optimizing physical therapy-based treatments for patellar tendinopathy. This systematic review focuses on three commonly employed interventions: dry needling, isometric exercises, and eccentric exercises. By critically evaluating the current evidence, we aim to provide insights into the most effective physical therapy strategies for managing this challenging condition in athletes (11, 12).

The primary purpose of this systematic review is to evaluate the effects of dry needling, isometric exercises, and eccentric exercises on pain severity and functional impairment in individuals diagnosed with patellar tendinopathy. The primary outcome measure is the reduction in pain severity, assessed through validated tools such as the Visual Analog Scale (VAS) and the Numeric Rating Scale (NRS). The secondary outcome measure is the improvement in functional capacity**,** evaluated using the Victorian Institute of Sports Assessment-Patella (VISA-P) questionnaire, which measures symptoms, physical function, and sports-related performance. This review aims to provide a comprehensive analysis of the effectiveness of these interventions in treating patellar tendinopathy, particularly among athletes.

## Methods

Detailed Description of Physical Therapy Treatments for Patellar Tendinopathy.

### Dry needling

Dry needling for patellar tendinopathy involves inserting thin, solid filament needles into specific points of the patellar tendon. The procedure typically follows these steps: the patient lies supine with the knee slightly flexed. The practitioner then palpates the patellar tendon to identify areas of tenderness or nodules. Using aseptic technique, the skin is cleaned with an antiseptic solution. A sterile, single-use needle is inserted into the identified area, often guided by ultrasound for precision. The needle may be rotated or moved up and down to elicit a local twitch response. The needle is left in place for a short period, usually between 10 and 30 s, before removal. This process may be repeated at different points along the tendon (13).

Dry needling is believed to work through several mechanisms. Mechanically, the needle may disrupt adhesions or scar tissue within the tendon. Biochemically, needle insertion may stimulate the release of endogenous opioids and other substances that modulate pain. Neuromuscularly, it may alter muscle tone and trigger point activity in the surrounding musculature. Additionally, the microtrauma caused by needling may promote local vasodilation and increase blood flow to the tendon. Physiologically, dry needling is thought to stimulate a localized healing response by creating controlled microtrauma. This may initiate a cascade of cellular events, such as the release of growth factors and recruitment of fibroblasts, potentially promoting tendon remodelling and repair (14).

### Isometric exercises

Isometric exercises for patellar tendinopathy typically involve sustained contractions of the quadriceps muscle without joint movement. A common protocol for these exercises includes positioning the patient seated with the knee flexed at a specific angle, often 60°. The patient uses a leg extension machine or resistance band to perform a maximal or submaximal quadriceps contraction against immovable resistance. The contractions are held for 30–45 s, with 5 sets commonly performed. This protocol is often done daily or several times per week. Isometric exercises may activate descending pain inhibitory systems, reduce cortical inhibition to improve motor output, stimulate tendon remodelling through controlled loading, and improve quadriceps activation and control (6).

The rationale behind isometric exercises is that they provide a means of loading the tendon in a controlled manner, potentially stimulating adaptive responses without creating excessive shear forces. The sustained nature of the contraction may have specific effects on pain processing pathways, offering both immediate and longer-term pain relief.

### Eccentric exercises

Eccentric exercises for patellar tendinopathy focus on the lengthening phase of muscle contraction. A typical protocol involves standing on a 25° decline board to increase the load on the patellar tendon. The patient performs a single-leg squat, lowering the body slowly over 3–4 s. To return to the starting position, the patient uses the non-affected leg or arms, minimizing concentric load on the affected side. The typical protocol involves 3 sets of 15 repetitions. As tolerance improves, weight is added, such as a backpack with weights. These exercises are often performed twice daily, 7 days a week (11).

Eccentric loading during these exercises may stimulate collagen synthesis and improve tendon structure. Additionally, it may increase the length and compliance of the muscle-tendon unit, reduce pain sensitivity in the tendon through progressive loading, and improve motor control and coordination. The physiological rationale for eccentric exercises is that they subject the tendon to high loads during the lengthening phase, which more closely mimics the demands placed on the tendon during jumping activities. This controlled overload may stimulate adaptive responses in the tendon, improving its capacity to withstand and transmit force (8).

The Preferred Reporting Items for Systematic Review and Meta-Analyses (PRISMA) statements (15, 16) were used as the article's guidelines.

### Search strategy

To ensure a comprehensive review of the literature, F.S. and D.M. search strategy in response to feedback. The initial search included studies published between 2010 and 2022. However, recognizing that physical therapy treatments for patellar tendinopathy have been studied for several decades, we extended our search to include studies published from 1990 onwards. This expanded timeframe allows to capture the evolution of these treatments and provide a more comprehensive overview of the existing evidence.

F.S. and D.M. conducted a thorough search of the following electronic databases: PubMed, Cochrane Library, PEDro, EMBASE, CINAHL, SPORTDiscus, Web of Science, and Scopus. The search was last updated on September 2022.

F.S. and D.M. used the following search terms and their combinations:

For the condition: “patellar tendinopathy” OR “jumper's knee” OR “patellar tendinitis” OR “patellar tendinosis”

For treatments: “dry needling” OR “acupuncture” OR “isometric exercise” OR “isometric training” OR “eccentric exercise” OR “eccentric training” OR “physical therapy” OR “physiotherapy” OR “rehabilitation”

F.S. and D.M. also used database-specific controlled vocabulary (e.g., MeSH terms in PubMed) to enhance the sensitivity of our search.

In addition to electronic database searches, A.S. and D.M. manually screened the reference lists of included studies and relevant systematic reviews to identify any additional eligible studies. A.S. and D.M. also searched clinical trial registries, including clinicaltrials.gov and the WHO International Clinical Trials Registry Platform, to identify ongoing or recently completed studies that may not yet be published.

To further ensure comprehensiveness, F.S. and A.S. contacted experts in the field of sports medicine and physical therapy to inquire about any unpublished or ongoing studies relevant to our review.

### Eligibility criteria

The eligibility criteria for this systematic review were defined using the PICO (Population, Intervention, Comparison, Outcome) framework (17).

### Inclusion criteria

The following articles were considered eligible for inclusion in this review based on the outlined criteria:

1. Type of Study: The review focused on randomized controlled trials (single- or double-blinded) or cross-over trials. Studies were considered regardless of whether they were open-access or paid, provided they contained published evidence relevant to the topic.
2. Type of Participant: Participants included in the studies had to have a clinically or imaging-confirmed diagnosis of patellar tendinopathy, with a history of knee pain localized to the patellar tendon for a minimum duration of three months, affecting one or both knees. Eligible participants were required to be at least 16 years of age, with consistent follow-up, and to have practiced volleyball or basketball at least three times per week.
3. Type of Intervention: Studies investigating physiotherapy interventions involving isometric exercises, eccentric exercises, or dry needling were included. Physical therapy exercises or adjunct procedures were also considered, as these conservative treatment strategies are frequently employed in conjunction with the primary interventions.
4. Comparison/Control: Where applicable, studies that included a comparison or control group were included, and the details of these comparisons were documented according to the study protocols.
5. Type of Outcome Measure: The primary outcome measure of interest was the assessment of pain severity using the Visual Analogue Scale (VAS) (18) or Numeric Rating Scale (NRS). Both tools rate pain on a scale from 0 to 10, with 0 indicating no pain and 10 representing the worst possible pain. The secondary outcome of interest was functional impairment, which was evaluated using the Victorian Institute of Sports Assessment-Patella (VISA-P) questionnaire. The VISA-P measures symptoms, function, and athletic ability, with scores ranging from 0 (severe impairment) to 100 (fully active and symptom-free). These measures were used to assess pain levels and functional capabilities in individuals with patellar tendinopathy (19, 20).

### Exclusion criteria

Studies comparing various types of therapies, such as surgical interventions or injection-based treatments, were excluded from this review. Additionally, research not published in English, lacking full-text availability, or not involving patients diagnosed with patellar tendinopathy was omitted. Studies involving participants with cardiovascular disease, rheumatoid arthritis, septic arthritis, or other inflammatory arthritic conditions were excluded to avoid confounding factors from other chronic diseases. Furthermore, observational studies, recommendations, narrative reviews, surveys, and expert commentaries were not included (21, 22).

## Data collection and analysis

### Selection of studies

Two independent reviewers (D.M. and A.S.) screened the titles and abstracts of identified articles to determine their eligibility for inclusion in the review. Full texts of potentially relevant studies were then obtained for further evaluation. The reviewers remained blinded to the authors, institutions, publication sources, and research outcomes during the selection process. Each manuscript was independently reviewed and assessed by an expert panel. Any disagreements between the reviewers regarding study inclusion were resolved through discussion.

### Data extraction

Two independent reviewers (F.S. and D.M.) extracted relevant data from the included studies. Information such as author, year of publication, journal, inclusion criteria, participant demographics (age, sex), duration of treatment, rehabilitation plans, outcome measures, and risk of bias were recorded. A standardized Microsoft Excel spreadsheet specifically designed for clinical trials was used to systematically collect the data. In cases of ambiguous or missing data, attempts were made to contact the authors of the respective studies for clarification. Any discrepancies were resolved through discussion with the authors.

### Evaluation of methodological quality

The methodological quality of each included trial was assessed using the Physiotherapy Evidence Database (PEDro) scale, based on the Delphi list. The PEDro scale consists of 11 items, including eligibility criteria, random allocation, allocation concealment, baseline group comparability, blinding of subjects, therapists, and assessors, use of appropriate timing protocols, intention-to-treat analysis, between-group comparisons, point estimates, and data variability. The scale ranges from 1 to 10, with higher scores indicating greater methodological rigor. For this review, studies scoring less than five were classified as low-quality evidence, while those scoring five or higher were considered high-quality evidence.

### Risk of bias assessment

The McMaster Critical Review Form for Quantitative Studies was used to assess the risk of bias across the included studies. This tool provides a structured approach to evaluating studies, with responses of “Yes,” “No,” or “Not addressed,” which are then scored based on the criteria met. This tool was selected for its widespread use, availability, and ability to assess a variety of study designs. Two reviewers independently evaluated the level of evidence and grade of recommendation for each study, utilizing the Oxford Centre for Evidence-Based Medicine grading system (23). Disagreements between the reviewers were resolved through the involvement of a third reviewer, though this was not required in practice.

### Summary measures

For continuous data, results were reported as mean differences, with 95% confidence intervals and *p*-values. For categorical data, such as VAS or VISA scores, percentage changes, 95% confidence intervals, *p*-values, and chi-squared values were provided when available.

### Data synthesis

Attempts were made to pool data for meta-analysis; however, due to the heterogeneity of the included studies and the limited number of trials, this was not feasible. Additionally, time constraints precluded the completion of a meta-analysis. Conclusions were instead derived from the best available evidence synthesis, which took into account the methodological quality of the studies, their findings, and the consistency of the results across trials. This study was registered with PROSPERO (number CRD42022360057).

### Study selection

**Figure 1:**
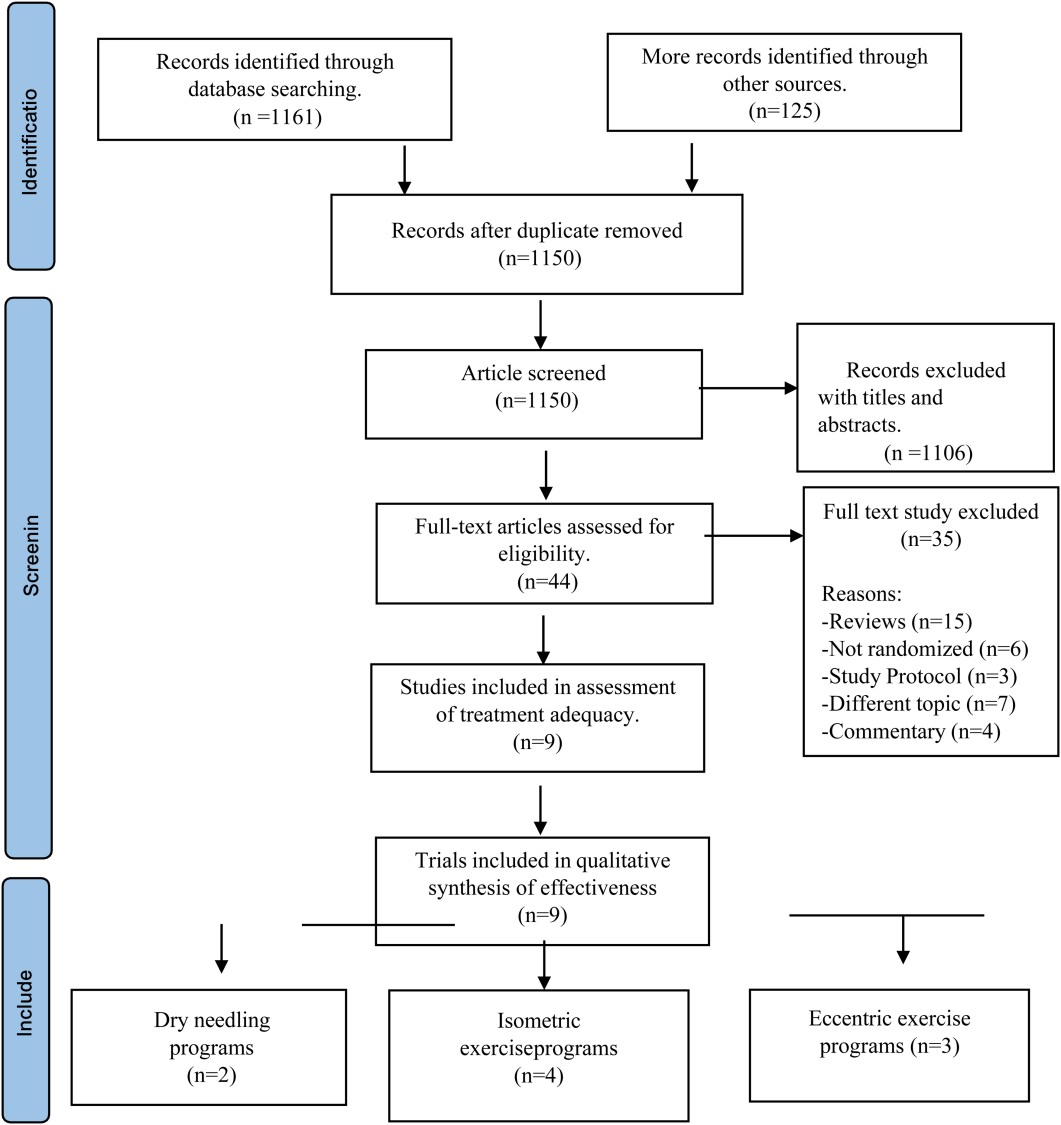
Flowchart of included clinical trials and study selection.

An initial search yielded 1,150 articles for potential inclusion. After removing duplicates and screening titles and abstracts, 1,106 studies were excluded, as shown in Figure 1. The full texts of the remaining 44 studies were evaluated for eligibility, leading to the exclusion of 35 articles. The reasons for exclusion were as follows: 15 articles were reviews, 6 were non-randomized studies, 3 described study protocols, 7 focused on different topics, and 4 were commentaries. Ultimately, 9 articles (10–12, 24–29) were included in the systematic review to assess the therapeutic effectiveness of dry needling, isometric exercises, and eccentric exercises for patellar tendinopathy.

### Characteristics of included studies

Table 1 summarizes the essential characteristics of the nine included clinical trials (10–12, 24–29). Two independent reviewers extracted data from each study. All included studies were published in English and focused on non-surgical interventions for treating patellar tendinopathy in athletes. In total, 186 participants were included in the eccentric exercise programs (10, 11, 29), 69 participants in dry needling programs (12, 25), and 75 participants in isometric exercise programs (24, 26–28). All trials involved participants aged between 16 and 48 years, with confirmed diagnoses of patellar tendinopathy. Each study assessed two primary outcomes: pain severity and functional improvement.

**Table 1:** Characteristics of included studies.

Author [year]	Study design and blinding type	Participant characteristics	Interventions	Co-intervention	Comparison (if any)	Evaluation outcomes	Follow-up sessions	Conclusion of the study
Dragoo et al. [2014] (25)	Double blinded RCT	*N*: 21 (20 M, 1 F) Age: (35 ± 13) Sports played: volleyball and basketball	US guided dry needling (DN) + Eccentric exe (*N*: 12) 26 weeks	Leukocyte-rich (PRP) + Eccentric exe (*N*: 9	None	(1) Pain: (VAS) DN group showed significant improvement at ≥26 wk. (*p* = 0.02). Wk 12: DN 2.3 ± 1.6 [CI (1.4–3.2)] PRP 1.7 ± 1.7 [CI (0.5–2.8)]. Wk 26: DN 0.3 ± 0.5 [CI (0.0–0.7)] PRP 1.7 ± 1.5 [CI (0.6–2.8)]. (2) Function: (VISA-P) DN group was seen to have net improvement (*p* = 0.0001). Wk 12: DN 52 ± 20.3 [CI (40.0–64.0)] PRP 66.4 ± 20.2 [CI (53.2–79.7)]. Wk 26: DN 83.9 ± 9.0 [CI (78.0–89.8)] PRP 67.8 ± 21.9 [CI (53.4–82.1)].	Pre-Tx, 3, 6, 9, 12 and ≥ 26 wk.	VISA-P: The eccentric ex. +DN group showed only slight improvement at 12 weeks but showed higher improvement at 26 weeks. At 12 weeks, the group with eccentric ex. +PRP + DN had improved more than the other group. By week 26, there had been no statistically significant difference between the two groups, and both had shown clinically significant improvement. VAS: At 12 and 26 weeks, there was no discernible difference between the groups.
Lopez- Royo et al. [2021] (12)	Double blinded RCT	*N*: 48 (42 M, 6 F) Age: 32.46 yr Sports played: any sports	Dry needling + Eccentric exe (*N*: 16)	(1) Percutaneous needle electrolysis (PNE) + Eccentric exe (*N*: 16) (2) Control group sham needle (*N*: 16)	None	(1) Pain: (VAS) DN + EE group resulted in decrease in pain at 22 wk. (*p* ≤ 0.05). Wk 10: DN + EE 2.5 [CI (1.1–3.9)] PNE + EE 2.8 [CI (1.8–3.7)]. Wk 22: DN + EE 0.9 [CI (0.3–1.5)] PNE + EE 2 [CI (0.8–3.2)]. (2) Function: (VISA-P) effect was found in both group at 10- and 22 wk. (*p* = 0.01). Wk 10: DN + EE 73.9 [CI (63.9–83.8)] PNE + EE 65.7 (CI [56.6–74.8). Wk 22: DN + EE 78.2 [CI (66.2–90.1)] PNE + EE 73.3 [CI (62.1–84.5)].	Pre-Tx, 10 and 22 wk	DN or PNE combined with an EE program has not shown to be more effective than a program of only EE to improve disability and pain in patients with PT in the short (10 wk) and medium (22 wk) terms. Clinical improvements were not associated with structural changes in the tendon
Rio et al. [2015] (24)	Single blinded cross-over trial	*N*: 6 (6 M, 0 F) Age: 26.9 (18–40 yr) Sports played: volleyball	Isometric group Biodex Pro: 5 sets × 45-s at 60° Knee Flex at 70% MVIC	None	Isotonic group Leg extension machine: 4 sets × 8 reps with 4- s EP and 3-s CP at 100% 8RM	(1) Pain: (NRS) significant pain reduction in both groups, isometric group (*p* = 0.004) and isotonic group (*p* = 0.04), 45 min post-Tx, pain reduction still in isometric group (*p* < 0.001) but not in isotonic (*p* > 0.05). (2) Function: (VISA) post-Tx not reported	Pre-Tx, immediately post-Tx, 45 min post-Tx	Isometric exercise leads in significantly reduced tendon discomfort instantly and the reduction in pain was sustained when assessed at 45 min post exercise. When assessed 45 min after exercise, isotonic exercise did not produce the same level of pain reduction, nor did the pain reduction last.
Rio et al. [2016] (26)	Single blinded RCT	*N*: 20 (18 M, 2F) Age: more than 16 yr Sports played: elite and sub elite volleyball and basketball	Isometric group Leg extension machine: 5 sets × 45-s at 60° Knee Flex at 80% MVIC	None	Isotonic group Leg extension machine: 4 sets × 8 reps with 4- s EP and 3-s CP at 80% 8RM	(1) Pain: (NRS) both groups had reduction in pain, more decline in pain seen in isometric group (mean change = 1.8 ± 0.39) than isotonic group (mean change = 0.9 ± 0.25). (2) Function: (VISA) both groups improved	Pre-Tx, immediately post-Tx and 4wk	Both protocols appear efficacious for in-season athletes to reduce pain, however, isometric contractions demonstrated significantly greater immediate analgesia throughout the 4-week trial. Greater analgesia may increase the ability to load or perform.
Van Ark et al. [2016] (27)	Single blinded RCT	*N*: 29 (27 M, 2 F) Age: 23 ± 4.7 (16–32 yr) Sports played: volleyball and basketball	Isometric group (*N*:13) Leg extension machine: 5 sets × 45-s at 60° Knee Flex at 80% MVIC	None	Isotonic group (*N*: 16) Leg extension machine: 4 sets × 8 reps with 3- s CP and 4-s EP at 80% 8RM	(1) Pain: (NRS) slight improvement in pain at 4 wk for isometric group (*p* = 0.012). (2) Function: (VISA-P) greater change for isometric group at 4 wk (*p* = 0.028).	Pre-Tx and 4wk	Both isometric and isotonic exercise programs are easy-to-use exercises that can reduce pain from patellar tendinopathy for athletes in-season.
Holden et al. [2019] (28)	Single blinded cross-over trial	*N*: 20 (12 M, 8 F) Age: (18–40 yr) Sports played: handball, volleyball, gymnastics, and triathlon	Isometric group Biodex 4 Pro: 5 sets × 45-s at 60° Knee Flex at 70% MVIC	None	Dynamic resistance group Leg extension machine: 3 set × 8 reps with 3-s CP and 3-s EP at 80% 8RM	(1) Pain: (NRS) no statistically significant difference among two groups were observed. (2) Function: (VISA-P) at the conclusion of treatment, there was little difference in the level of improvement	Pre-Tx, immediately post-Tx, 45 min post-Tx	While patients with patellar tendinopathy decreased pain during SLDS in response to resistance training, but the magnitude was small. Contraction mode may not be the most important factor in determining the magnitude of pain-relieving effects. Similarly, there were only small increases in PPTs at the tibialis anterior which were not superior for isometric exercise.
Dimitrios et al. [2012] (29)	Controlled clinical trial	*N*: 43 (31 M, 12 F) Age: 26.38 (18–30 yr) Sports played: Not specified	Eccentric training group: 3 sets × 15 rep single leg squat at 25° decline board	None	Eccentric training group with static stretching for 30-s	(1) Pain: (VISA-P) both groups showed decline in pain. (2) Function: (VISA-P) there was a significant improvement in both groups but more in eccentric + static stretching group (*p* < 0.0005). Wk 4: Eccentric + stretching group +42 units [CI (33.3–48.6)] Eccentric group + 28 units [CI (24.4–33.5)] Wk 24: Eccentric + stretching group +50 units [CI (38.9–54.5)] Eccentric group +31 units [CI (26.8–36.1)]	Pre-Tx, 4 and 24 wk	Eccentric training and static stretching exercises is superior to eccentric training alone to reduce pain and improve function in patients with patellar tendinopathy at the end of the treatment and at follow-up.
Breda et al. [2022] (11)	Single blinded RCT	N: 76 (58 M, 9 F) Age: (18–35 yr) Sports played: volleyball, soccer, basketball, and handball	Progressive tendon-loading exercise therapy (PTLE) or eccentric exercise therapy (EET) for 24 weeks	None	Progressive tendon-loading exercise group (1) Isometric static exe (2) Isotonic (dynamic) exe (3) Plyometric exe (4) Sport- specific exe	Function: (VISA-P) at 24 weeks, the PTLE group had statistically significant results (*p* < 0.001).	Pre-Tx, 12 and 24 wk	Patellar tendon stiffness, assessed with shear-wave elastography, is unsuitable to use as a single predictive measurement for clinical outcome. Decreasing stiffness during exercise therapy is associated with improved clinical outcome in athletes recovering from patellar tendinopathy.
Breda et al. [2021] (10)	Single blinded RCT	*N*: 76 (58 M, 18 F) Age: (18–35 yr), Sports played: Not specified	Eccentric exe group 3 sets × 15 reps single leg squat and sport specific exe	None	Progressive tendon-loading exercise group (1) Isometric static exe (2) Isotonic (dynamic) exe (3) Plyometric exe (4) Sport- specific exe	Function: the improvement in VISA-*P* score was significantly better for PTLE than for EET after 24 weeks (28 vs. 18 points, adjusted mean between-group difference, 9 (95% CI 1–16); *p* = 0.023	Pre-Tx, 12 and 24 wk	In patients with PT, PTLE resulted in a significantly better clinical outcome after 24 weeks than EET. PTLE are superior to EET and are therefore recommended as initial conservative treatment for PT.

Representation in Table 5.

*N*: number of participants; M: Male; F: Female; Yr: Years; Exe: exercise; RCT: randomized controlled trial; VISA: Victorian Institute of Sports Assessment; VAS: Visual Analogue Scale; US: ultrasound; NRS: Numeric Rating Scale; RM: repetition maximum; CP: concentric phase; EP: eccentric phase; MVIC: maximal voluntary isometric contraction; Reps: repetitions; Pre-Tx: pre-treatment; Post-Tx: Post-Treatment; wk: week; min: minute; CI: confidence interval.

Based on Sackett's level of evidence, six of the nine studies achieved a Level Ⅱ ranking (10–12, 25–27), while the remaining three received a Level Ⅲ ranking (24, 28, 29), as indicated in Table 1. Most participants were regular athletes, with basketball and volleyball being the most studied sports. The follow-up periods varied among the studies. Three trials followed participants for up to 24 weeks (10, 11, 29), while others followed up at shorter intervals, such as immediately post-intervention and 45 min later (24, 26, 28). One study had a follow-up period of 26 weeks (25), another 22 weeks (12), and one only 4 weeks (27).

### Methodological quality

The methodological quality of the included studies was evaluated using the PEDro scale, and the results are summarized in Table 2. One of the nine studies had already been assessed using the PEDro criteria in previous research (25). Two independent reviewers assessed the remaining eight studies (10–12, 24, 26–29). All studies received a score greater than 5 on the PEDro scale, indicating high methodological quality. Specifically, five studies received a score of 8 (10, 12, 24–26), while the other four received a score of 7 (11, 27–29). Overall, all nine studies were considered high-quality research and demonstrated effective interventions for reducing pain and improving functional impairment associated with patellar tendinopathy.

**Table 2:** Quality assessment according to PEDro scale.

Authors [year]	Eligibilty	Random allocation	Concealed allocation	Baseline comparability	Subject blinding	Therapist blinding	Assessor blinding	Less than 15% drop out	Intention to treat analysis	Statistical comparisons between groups	Data on point measures and variability	Final points (criteria one not summed)
Dragoo et al. [2014] (25)	Yes	Yes	Yes	No	Yes	Yes	No	Yes	Yes	Yes	Yes	8
Lopez- Royo et al. [2021] (12)	Yes	Yes	Yes	Yes	Yes	No	Yes	Yes	No	Yes	Yes	8
Rio et al. [2015] (24)	Yes	Yes	Yes	Yes	Yes	No	No	Yes	Yes	Yes	Yes	8
Rio et al. [2016] (26)	Yes	Yes	Yes	Yes	Yes	No	No	Yes	Yes	Yes	Yes	8
Van Ark et al. [2016] (27)	Yes	Yes	Yes	Yes	Yes	No	No	Yes	No	Yes	Yes	7
Holden et al. [2019] (28)	Yes	Yes	Yes	Yes	Yes	No	No	Yes	No	Yes	Yes	7
Dimitrios et al. [2012] (29)	Yes	Yes	No	Yes	Yes	No	Yes	Yes	No	Yes	Yes	7
Breda et al. [2022] (11)	Yes	Yes	No	Yes	Yes	No	Yes	No	Yes	Yes	Yes	7
Breda et al. [2021] (10)	Yes	Yes	Yes	Yes	Yes	No	No	Yes	Yes	Yes	Yes	8
Sub-Item score	9	9	7	8	9	1	5	8	4	9	9	

### Assessment of risk of bias and level of evidence

We used the McMaster Critical Review Form for Quantitative Studies to enhance the quality of our systematic review Table 3. The overall average score ranged from 70% to 93%. Four clinical trials showed moderate quality (at least 75% of an average), three studies demonstrated (78%), and two studies were of excellent quality (at least 93%). As a result, the included studies generally provided evidence of moderate to high quality. Nine studies were included in this review Table 4. Oxford Centre for Evidence-based Medicine was taken into consideration while determining the level of evidence for each clinical study in Table 5. In terms of the level of evidence and grade of recommendation Table 6 (30), six studies were level 1B (individual randomized controlled trials with narrow confidence intervals), with the recommendation of grade A (strong recommendation that is expected to be followed, unless there are compelling reasons to deviate from the recommendation in an individual) (10–12, 25–27). Three studies were 2B (Individual cohort study (including low quality Randomized Controlled Trial), as two were single-blinded randomized cross-over trials (28, 31) and one was a controlled clinical trial (29), with recommendation of grade B (satisfactory recommendation), but these studies were still appropriate in evaluating the effects of the intervention.

**Table 3:** Summary of Key study details and methodological components.

Author [year]	Study purpose and aim	Background literature	Study design (according to McMaster Tool	Sample characteristics	Sample size	Reliable outcome measures	Valid outcome measures	Intervention details
Dry Needling								
Dragoo et al. [2014] (25)	Yes	Yes	Double blinded	Yes	Yes	Yes	Yes	Yes
			Randomized controlled trial					
Lopez- Royo et al. [2021] (12)	Yes	Yes	Double blinded	Yes	Yes	Yes	Yes	Yes
			Randomized controlled trial					
Isometric								
Rio et al. [2015] (24)	Yes	Yes	Single-blinded	Yes	No justification of sample size	Yes	Yes	Yes
			Randomized cross- over trial with two intervention arms					
Rio et al. [2016] (26)	Yes	Yes	Single-blinded	Yes	No justification of sample size	Yes	Yes	Yes
			Randomized clinical trial					
Van Ark et al. [2016] (27)	Yes	Yes	Single-blinded	Yes	No justification of sample size	Yes	Yes	Yes
			Randomized clinical trial					
Holden et al. [2019] (28)	Yes	Yes	Single-blinded cross-over trial	Yes	No justification of sample size	Yes	Yes	Yes
Eccentric								
Dimitrios et al. [2012] (29)	Yes	Yes	Controlled clinical trial	Yes	No justification of sample size	Yes	Yes	Yes
Breda et al. [2022] (11)	Yes	Yes	Double blinded	Yes	No justification of sample size	Yes	Yes	Yes
			Randomized clinical trial					
Breda et al. [2021] (10)	Yes	Yes	Double blinded	Yes	No justification of sample size	Yes	Yes	Yes
			Randomized clinical trial					

**Table 4:** Level of evidence and grade of recommendation.

Study	Level of evidence	Design	Level of recommendation
Dry needling			
Dragoo et al. (25)	1B	Individual Randomized Controlled Trial	A
López-Royo et al. (12)	1B	Individual Randomized Controlled Trial	A
Isometrics			
Rio et al. (31)	2B	Individual cohort study (including low quality Randomized Controlled Trial)	B
Rio et al. (26)	1B	Individual Randomized Controlled Trial	A
Van Ark et al. (27)	1B	Individual Randomized Controlled Trial	A
Holden et al. (28)	2B	Individual cohort study (including low quality Randomized Controlled Trial)	B
Eccentric			
Dimitrios et al. (29)	2B	Individual cohort study (including low quality Randomized Controlled Trial)	B
Breda et al. (11)	1B	Individual Randomized Controlled Trial	A
Breda et al. (10)	1B	Individual Randomized Controlled Trial	A

**Table 5:** Levels of evidence (Oxford centre for evidence-based medicine) (30).

Level	Therapy/prevention, etiology/harm
1a	Systematic review (with homogeneity) of randomized controlled trials
1b	Individual randomized controlled trial (with narrow confidence interval)
1c	All or none
2a	Systematic review (with homogeneity) of cohort studies
2b	Individual cohort study (including low quality randomized controlled trial, e.g., <80% follow-up)
2c	“outcomes” research; ecological studies
3a	Systematic review (with homogeneity) of case-control studies
3b	Individual case-control Study
4	Case-series (and poor-quality cohort and case-control studies)
5	Expert opinion without explicit critical appraisal, or based on physiology, bench research or “first principles.”

**Table 6:** Grades of recommendation (Oxford centre for evidence-based medicine) (30).

Grade	Contents
A	consistent level 1 studies
B	consistent level 2 or 3 studies or extrapolations from level 1 studies
C	level 4 studies or extrapolations from level 2 or 3 studies
D	level 5 evidence or troublingly inconsistent or inconclusive studies of any level

### Results of individual studies

The process of study selection is illustrated in Figure 1.

An initial search yielded 1,150 articles for potential inclusion. After removing duplicates and screening titles and abstracts, 1,106 studies were excluded. The full texts of the remaining 44 studies were evaluated for eligibility, leading to the exclusion of 35 articles. The reasons for exclusion were as follows: 15 articles were reviews, 6 were non-randomized studies, 3 described study protocols, 7 focused on different topics, and 4 were commentaries. Ultimately, 9 articles (10–12, 24–29) were included in the systematic review to assess the therapeutic effectiveness of dry needling, isometric exercises, and eccentric exercises for patellar tendinopathy.

Tables 7 and 8 summarizes the conclusions of all trials, comparing the effects on pain severity and functional impairment.

**Table 7:** Risk of bias in individual studies.

Author [year]	Contamination avoided	Co-intervention avoided	Statistical significance	Appropriate analysis method	Clinical importance	Drop-outs	Appropriate conclusion	Total score
Dry needling								
Dragoo et al. [2014] (25)	Yes	No, not withdrawn from sports	Yes	Yes	Yes	Yes, reported	Yes	13/14
Lopez-Royo et al. [2021] (12)	Yes	No, not withdrawn from sports	Yes	Yes	Yes	Yes, reported	Yes	13/14
Isometric								
Rio et al. [2015] (24)	NA	No, not withdrawn from sports	Yes	Yes	Yes	No Dropouts	Yes	10/13
Rio et al. [2016] (26)	Yes	No, not withdrawn from sports	Yes	Yes	Yes	Yes, reported	Yes	12/14
Van Ark et al. [2016] (27)	Yes	No, not withdrawn from sports	Yes	Yes	Yes	No Dropouts	Yes	11/14
Holden et al. [2019] (28)	Not addressed	No, not withdrawn from activity	No	Yes	Not addressed	Yes, reported	No	8/14
Eccentric								
Dimitrios et al. [2012] (29)	Not addressed	No, not withdrawn from activity	Yes	Yes	Yes	No Dropouts	Yes	10/14
Breda et al. [2022] (11)	Not addressed	Not addressed	Yes	Yes	Yes	Yes, reported	Yes	11/14
Breda et al. [2021] (10)	Not addressed	Yes	Yes	Yes	Yes	Yes, reported	Yes	12/14

**Table 8:** Results of studies included in subgroup analysis.

Authors	Comparator	Pain severity	Functional improvement
Dry needling			
Dragoo et al. [2014] (25)	None	Strong	Strong
Lopez-Royo et al. [2021] (12)	None	Strong	Strong
Isometric			
Rio et al. [2015] (24)	Isotonic exercise	Strong	Not reported
Rio et al. [2016] (26)	Isotonic exercise	Strong	Weak
Van Ark et al. [2016] (27)	Isotonic exercise	Strong	Weak
Holden et al. [2019] (28)	Dynamic resistance exercise	Weak	Weak
Eccentric			
Dimitrios et al. [2012] (29)	Eccentric exercise with stretching	Strong	Strong
Breda et al. [2022] (11)	Eccentric exercise	Strong	Strong
Breda et al. [2021] (10)	Eccentric exercise	Strong	Strong

Representation in table; Strong: Significant improvement; Weak: lesser improvement than comparator.

## Dry needling

Two clinical trials, Dragoo et al. (2014) and Lopez et al. (2021), evaluated the efficacy of dry needling under ultrasound guidance to treat pain and functional limitations caused by patellar tendinopathy (12, 25).

• In Dragoo et al. (2014), a radiologist used an ultrasound probe to deliver 10 insertions into the patellar tendon region over a series of sessions (25). The results demonstrated significant improvement in pain, with a reduction in the VAS (Visual Analogue Scale) score over 26 weeks (*p* = 0.02), and a notable recovery in function measured by the VISA (Victorian Institute of Sports Assessment) score (*p* = 0.0001).
• Lopez et al. (2021) conducted a clinical trial involving the insertion of 3 needles across four sessions, resulting in 12 total insertions (12). The trial revealed a significant decrease in VAS pain severity by 22 weeks (*p* ≤ 0.05), with a corresponding improvement in functional ability as reflected in the VISA score.

## Isometric exercises

Four studies—Rio et al. (2015), Rio et al. (2016), Van Ark et al. (2016), and Holden et al. (2019)—examined the efficacy of isometric exercises in athletes with patellar tendinopathy, particularly those engaged in basketball or volleyball during the season (24, 26–28).

• Rio et al. (2015) implemented isometric exercises using either a Biodex or leg extension machine. Participants performed the exercises at 70%–80% of their maximum voluntary contraction at 60° knee flexion, holding for five sets of 45 s. The study reported significant pain reduction in the isometric group immediately after the intervention (*p* = 0.004) and maintained this reduction even 45 min post-intervention, while the isotonic group did not experience the same sustained pain relief (32).
• Rio et al. (2016) followed a similar protocol, showing that while both the isometric and isotonic exercise groups experienced a reduction in pain, the isometric group saw a greater reduction in NRS (Numeric Rating Scale) pain scores (1.8 ± 0.39) compared to the isotonic group (0.9 ± 0.25) (26).
• Van Ark et al. (2016) reported a modest reduction in pain during a 4-week intervention (27). Both Rio et al. (2016) and Van Ark et al. (2016) evaluated functional improvement and found that the VISA score significantly improved in both groups after 4 weeks, but with no significant difference between the isometric and isotonic groups.
• Holden et al. (2019) concluded that isometric exercises showed no substantial change in either pain severity or functional impairment by the end of the intervention (28).

## Eccentric exercise

Three clinical trials—Dimitrios et al. (2012), Breda et al. (2022), and Breda et al. (2021)—evaluated the effectiveness of Eccentric Exercise Therapy (EET) for managing patellar tendinopathy (10, 11, 29).

• Dimitrios et al. (2012) compared eccentric training to eccentric training combined with static stretching (29). The eccentric exercise method primarily involved performing 25° decline squats, consisting of three sets of 15 repetitions, where the concentric phase was executed with the non-affected leg and the eccentric phase was performed with the affected leg. The study reported a significant increase in the VISA-P (Victorian Institute of Sports Assessment - Patella) score at 4 weeks (*p* < 0.0005), indicating a reduction in pain and an enhancement in function towards the end of the treatment.
• Breda et al. (2022) and Breda et al. (2021) compared EET to Progressive Tendon-Loading Exercise (PTLE). PTLE involved four stages:
  ○ Stage 1: Daily isometric exercises, including leg extensions with 5 repetitions of 45 s at mid-range (60° knee flexion), held at 70% of maximum voluntary contraction.
  ○ Stage 2: The isometric exercises from Stage 1 were performed on alternate days, combined with new isotonic exercises on the second day. The isotonic exercises included leg extensions with 4 sets of 15 repetitions between 10° and 60° of knee flexion, which progressively increased.
  ○ Stage 3: Plyometric exercises, such as running.
  ○ Stage 4: Sports-specific exercises, tailored for activities such as basketball and volleyball.

Both studies assessed pain and functional improvement using the VISA-P questionnaire. Breda et al. (2022) observed improvement in clinical outcomes at 12 weeks (*p* = 0.02), while Breda et al. (2021) reported similar findings at 24 weeks (*p* < 0.001). However, both studies concluded that PTLE was superior to EET alone by the end of the treatment period.

## Discussion

Patellar tendinopathy is a common injury among recreational volleyball and basketball players. This systematic review aimed to evaluate the current evidence regarding the effects of dry needling, isometric exercises, and eccentric exercises on pain and function in individuals with patellar tendinopathy. To our knowledge, this is the first review focusing specifically on the clinical implications of physiotherapy-based interventions for managing this condition in athletes. All three interventions demonstrated successful outcomes in addressing pain and functional impairment.

The findings suggest that the results from isometric exercises and dry needling can be reliably used to guide clinical practice. In most clinical settings, the evidence from eccentric exercise can also be trusted according to the Oxford Centre for Evidence-Based Medicine (OCEBM) levels of evidence. Notably, two studies (Dragoo et al., 2014) and (Lopez et al., 2021) indicated that a therapeutic regimen combining exercise with platelet-rich plasma (PRP) injection alongside dry needling is more effective than exercise and ultrasound-guided dry needling alone. Similarly, the results of three studies evaluating eccentric exercises (Dimitrios et al., 2012; Breda et al., 2022; Breda et al., 2021) showed that eccentric training combined with static stretching is more beneficial than eccentric training alone, and that progressive tendon-loading exercises (PTLE) are superior to eccentric exercise training alone.

The McMaster tool's evaluation revealed that dry needling studies had a high quality, with an average score of 93%, while eccentric exercise programs scored an average of 78%. Isometric exercise studies had an average score of 75%, influenced by one moderate-quality study (Holden et al., 2019); however, overall, isometric studies yielded good results. A notable limitation of the current research is that many studies failed to specify sample sizes, and several did not adequately address whether contamination within control groups was avoided. These aspects present opportunities for improvement in future research.

Given that randomized controlled trials (RCTs) are the gold standard in medical research, the methodological quality of the nine trials included in this review was assessed using the PEDro scale. All studies scored above the cut-off of 5, with five trials achieving a score of 8/10 and four studies scoring 7/10.

Considering that patellar tendinopathy significantly impacts athletes' performance, treatment plans must prioritize pain control and functional enhancement, particularly during competitive seasons. One RCT indicated that isometric exercise was superior to both dry needling and eccentric exercise for alleviating acute pain. Specifically, Rio et al. (2015) demonstrated rapid analgesia in patients with patellar tendinopathy, evident just 45 min post-intervention. While dry needling also showed beneficial outcomes (Dragoo et al., 2014), pain relief was primarily observed at 26 weeks. Despite the extensive evidence supporting eccentric exercise programs, Breda et al. (2022) found that progressive tendon-loading exercise (PTLE) was more effective than eccentric exercise alone. Future research should compare the effects of different exercise modalities on pain in both short- and long-term contexts.

A previous review by Lim et al. (2018) suggested that isometric exercises are more effective for short-term pain relief compared to heavy slow resistance (HSR) or eccentric exercises. Similarly, a study by Challoumas (2021) concluded that isometric loading is effective for immediate pain relief, advocating for eccentric exercises as a first-line treatment for managing patellar tendinopathy. Additionally, this review found that dry needling demonstrated significant long-term effects, as it promotes analgesia and exerts mechanical effects on the tendon. These effects contribute to enhanced tensile strength and improved outcomes over time.

This systematic review rigorously adhered to robust methodologies to minimize bias. Firstly, the review process followed the Preferred Reporting Items for Systematic Reviews and Meta-Analyses 2020 (PRISMA) guidelines, ensuring external validity. Secondly, the studies were appraised based on the Oxford Centre for Evidence-Based Medicine Levels of Evidence and the PEDro scale, focusing on methodological quality and design applicability. Thirdly, subgroup evaluations were performed to assess clinical heterogeneity for result synthesis. Ultimately, nine clinical trials were analyzed to derive the review's outcomes.

A limitation of this systematic review is the inability to directly evaluate the effectiveness of isometric, eccentric, and dry needling interventions in isolation; thus, it remains unclear which treatment may be more effective for patellar tendinopathy. Furthermore, due to a scarcity of available evidence, this review relied on a limited number of free online publications rather than comprehensive access to all pertinent literature. Time constraints also precluded the possibility of conducting a meta-analysis, which could have provided deeper insights into the findings. Consequently, more thorough research is needed to clearly demonstrate the effectiveness of these interventions in treating patellar tendinopathy. Future studies should explore a broader range of physical therapy-based exercises and interventions to yield meaningful and reliable outcome measures.

## Conclusions

This systematic review evaluated the effectiveness of dry needling, isometric exercises, and eccentric exercises for managing pain and functional impairment in individuals with patellar tendinopathy. While dry needling and eccentric exercises demonstrated significant improvements in long-term pain reduction and function, especially in high-quality trials, the evidence remains limited by the small number of studies and varied methodologies. Isometric exercises showed short-term benefits in pain relief, particularly during athletic performance, but did not consistently improve long-term function across studies. Although these findings suggest that physical therapy-based interventions hold promise for treating patellar tendinopathy, the heterogeneity in study designs and outcome measures limits the ability to make definitive conclusions regarding the superiority of one treatment over another. More rigorous, high-quality randomized controlled trials are necessary to confirm these results and provide clearer guidance on the most effective treatment modalities for athletes with patellar tendinopathy.

## Data Availability

The original contributions presented in the study are included in the article/Supplementary Material, further inquiries can be directed to the corresponding author.
